# Preparation and Band Gap Characteristics of Composite Film/Substrate Instability System

**DOI:** 10.3390/ma15186248

**Published:** 2022-09-08

**Authors:** Huan Lv, Jiaming Deng, Yi Ren, Hao Zhang, Wang Zhang, Mangong Zhang, Haidong Liu, Bin Gu

**Affiliations:** 1 Key Laboratory of Testing Technology for Manufacturing Process, Ministry of Education, Southwest University of Science and Technology, Mianyang 621010, China; 2 Wuhan Second Ship Design and Research Institute, Wuhan 430064, China; 3 Tianjin Aerospace Relia Technology Co., Ltd., Tianjin 300462, China

**Keywords:** film/substrate system, instability, composite patterns, bandgap structures

## Abstract

Soft materials such as biological tissues are prone to deformation and generate different stable structures under external stimulation. This property is widely used to create tunable patterns, and the tuning of the wrinkling patterns can be applied to the control of elastic waves. In this paper, the wrinkling modes of film/substrate systems with different geometric dimensions and material parameters were studied. It is verified by numerical simulation that the elastic wave band gaps corresponding to the two wrinkling modes can be effectively superposed in one system, and the experimental samples with two wrinkling modes coexisting in one system were prepared by parameter optimization and a moisture-curing process. A vibration test showed that the hybrid system could effectively suppress the propagation of elastic waves. Combined with engineering needs, the wrinkling system under different loading conditions was studied, which provides a design guide for widening and regulating the elastic wave band gap.

## 1. Introduction

With the aging of the skin, the moisture content of each layer changes, thus generating compressive stress in the epidermis layer to wrinkle the skin [1,2,3,4,5,6]. Similar to this biological feature, soft metamaterials are sensitive to various external or internal stimuli due to their low elastic modulus and may undergo large deformation and local instability. Research on this wrinkling behavior has attracted considerable attention [4,5,7,8,9,10,11], and it has been proved that the characteristics of wrinkle patterns have found wide applications, including the manufacture of tunable patterns [12,13,14] and functional surfaces [15,16,17], as well as applications in acoustic concealment [18,19] and ultrasonic transducers [20,21], etc. In addition, periodic wrinkled patterns have become a research highlight in elastic wave control.

Alireza and Faramarz reported that a periodic surface pattern and corresponding stress could control elastic wave propagation in the low-thickness composite slab [22]. Wang et al. reviewed the band gap engineering, the abnormal behavior of wave propagation, and the tunable manipulation of waves based on different modulation mechanisms [23]. Li et al. demonstrated that the elastic wave band gap in the thin film/substrate bilayer system is largely dependent on the wrinkling mode [24]. Li and Cao further reported a new strategy to tune defect mode localization based on the special band structure of the film/substrate wrinkling system [25]. Sharma et al. developed an efficient gradient-based topology optimization method for maximizing the width of the band gaps [26]. Sharma et al. further indicated that a compressional longitudinal prestress and a lateral prestretch have a favorable impact on the widening of the frequency band gaps in soft compressible composites [27]. Therefore, metamaterials can be constructed by the periodic structure formed by instability and applied to elastic wave control. However, within the scope of existing research, the band gap width of these periodic wrinkling systems is limited and cannot well achieve the desired effect for engineering applications. The work of Li et al. showed that different wrinkling modes could coexist and evolve in a bilayer system, and elastic wave propagation behavior can be controlled by manipulating the composite wrinkling modes [24]. A wide band gap can be obtained by superimposing the band gaps of two single-instability topography film/substrate systems.

Previous research on the bilayer wrinkling system mainly focused on numerical simulation as there were great challenges in preparing bilayer wrinkling samples without warpage and debonding. In our recent work [28], we proposed a new manufacturing process to prepare the wrinkled film/substrate systems, which greatly diminished the residual stress and improved the interface bonding. However, the fabricated bilayer systems only have a uniform surface wrinkle wavelength and, thus, a limited band gap structure. In this paper, two groups of film/substrate wrinkling systems with wider band gaps were selected and combined to obtain a film/substrate system with composite instability morphology. Based on the general commercial software ABAQUS, (version 6.14, Dassault Systemes SIMULIA, Vélizy-Villacoublay, France), a finite element model was established to study the formation process of composite instability morphology. Furthermore, the effect of different parameters on the composite buckling morphology is examined to explore the band gap characteristics of the hybrid buckling morphology of the film/substrate system. Based on the moisture-curing process in the previous stage of this study [28], in which only a single pattern was induced, experimental samples with two wrinkling morphologies coexisting with one system were prepared, and vibration experiments were used to further explore the band gap characteristics of the film/substrate wrinkling system. The results show that the system has a wider band gap when two different instability patterns coexist in one system. When changing the combination parameters, the frequency and width of the band gap can be adjusted accordingly to obtain the band gap in need. In practical applications, we can further adjust the band gap structure by the external loading of the control system.

## 2. Finite Element Simulation

### 2.1. Model

**Figure 1:**
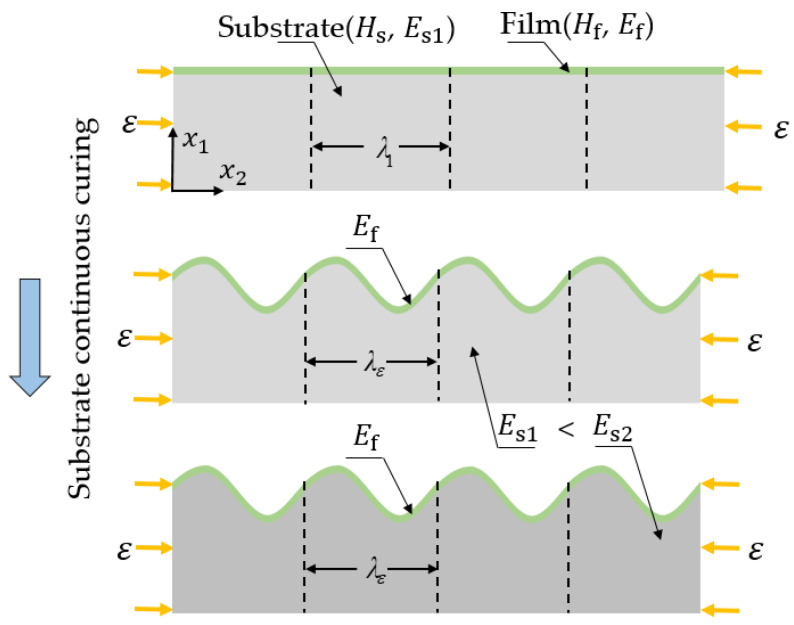
Schematic diagram of moisture-curing process.

In order to obtain the wrinkled samples without interfacial debonding and structural warping, we adopted the wet-curing process, as illustrated in Figure 1. The overall process is divided into two steps: (1) Controlling the time of curing when the substrate is in the plastic state, and the modulus of the substrate is $E_{s1}$. At this time, a hard film with a thickness of $H_{f}$ is attached to the soft substrate with a thickness of $H_{s\;}$. By applying the compressive strain *ε*, the bilayer system produces periodic wrinkle patterns. Each pattern with a length of  λ  is called a unit cell. (2) The compressive load is maintained until the substrate is completely cured, at which time the substrate modulus is expressed as $E_{s2}$ which is fixed as 3 MPa.

The critical wrinkle wavelength λ can be theoretically predicted by the equation below [25]:

(1) $$\lambda =2\pi H_{f}{\left(\frac{E_{f}}{3E_{s}}\right)}^{1/3}$$

When the external load continues to generate the corrugated structure, and until the substrate is completely cured, the length of each pattern is expressed as $\lambda_{\varepsilon }.$ As residual stress is greatly reduced by the second curing process, FE simulation of the wave propagation only inherits the geometric configuration after the surface morphology formation. The film and the substrate are modeled as neo-Hookean materials with different constitutive parameters and an element type of 8-node biquadratic plane strain quadrilateral composite, linear pressure, and reduced integration (CPE8RH). The governing equations and Bloch boundary conditions used in the calculation of energy band structures are consistent with those in reference [29] and are omitted for conciseness.

**Figure 2:**
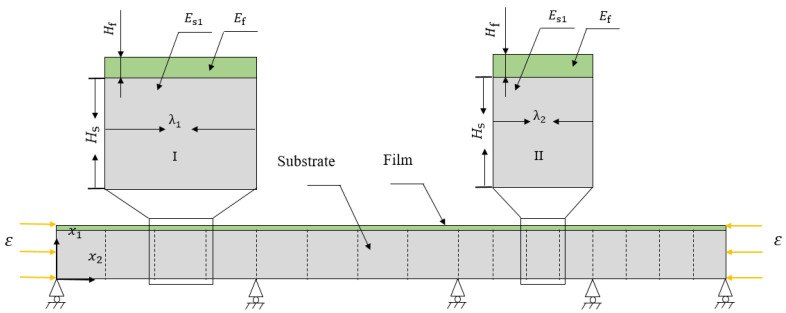
Finite element model hybrid film/substrate bilayer system.

When changing some parameters to construct different critical wrinkling wavelengths, we named the two different unit cells unit cell I and unit cell II, and their lengths are denoted as $\lambda_{1}$ and $\lambda_{2}$, respectively. The hybrid system established in our study consists of two types of periodic unit cells (I and II). Unit cells I and II are combined along the x_2_ direction, as shown in Figure 2. In addition, the basic design principles of wide band gaps needed to be considered; on the one hand, the band gap of the superimposed film/substrate system with two single morphologies is relatively wide, and on the other hand, there is an overlapping region in the frequency range between the two band gaps. The selection of parameters in subsequent studies is based on these two principles.

### 2.2. Wrinkling Patterns in the Composite Bilayer System

For this section, we simulated the formation of surface patterns through elastic instability to examine the effects of relevant parameters. The critical buckling analysis of the system was performed first, and then a critical buckling mode with a small factor (0.01) was introduced into the model as a geometric defect that triggers post-buckling. According to the buckling theory of the film/substrate system [30], the geometric dimension and material parameters can affect the surface wrinkle morphology. In this study, the case where the substrate is thick in Equation (1) was considered. Therefore, in this subsection, we systematically studied the influence of the thickness ratio, $H_{f}{/H}_{s}$, the modulus ratio, $E_{s1}{/E}_{s2}$, and the modulus ratio, $E_{f}{/E}_{s2}$, on the instability morphology and revealed the relationship between each parameter and the wrinkle wavelength and amplitude.

The film/substrate wrinkling systems with different thickness ratios were designed in combination to study the relationship between different thickness ratios and the hybrid instability morphology. Parameters are shown in Table 1, and the following parameters are kept unchanged: the compressive strain ε, substrate thickness $H_{s}$, the film modulus $E_{f}$, the substrate modulus at the first curing $E_{s1}$ and substrate modulus after full curing $E_{s2}$. The film thickness is from 0.6 to 1.2 mm, resulting in a thickness ratio from $\;3{.0\;\times \;10}^{-2}$ to $\;7{.0\;\times \;10}^{-2}$.

**Figure 3:**
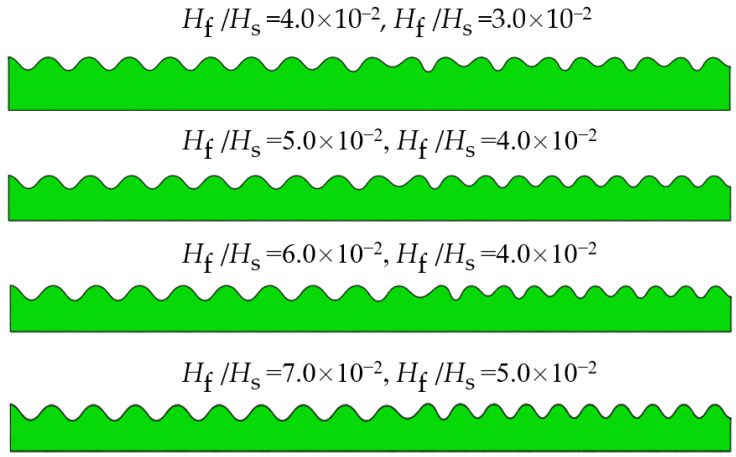
Influence of thickness ratio $H_{f}{/H}_{s}$ on the composite instability morphology.

Figure 3 shows the composite bilayer system with surface wrinkling patterns obtained by varying the thickness ratio, $H_{f}{/H}_{s}$, of the two periodic unit cells. Post-buckling analysis shows that when the thickness ratios are equal to $4{.0\;\times \;10}^{-2}$ and $3{.0\;\times \;10}^{-2}$, respectively, the composite system surface produces a sinusoidal pattern and a period-doubling pattern, and when the period-doubling pattern corresponds to a film thickness of 0.6 mm, it shows that the small thickness of the film is the main reason for the period-doubling mode. When the thickness ratios are taken as $5{.0\;\times \;10}^{-4}\;$ and $4{.0\;\times \;10}^{-4}$, respectively, the period-doubling pattern disappears, and the composite instability morphology consists of two sinusoidal patterns, but defects occur at the junction of the two patterns, which is caused by the mismatch of stress generated by the two patterns during the stabilization process. As the value between the two thickness ratios continues to increase, two sinusoidal patterns are also produced on the surface of the system, as well as defects in the joint. 

**Figure 4:**
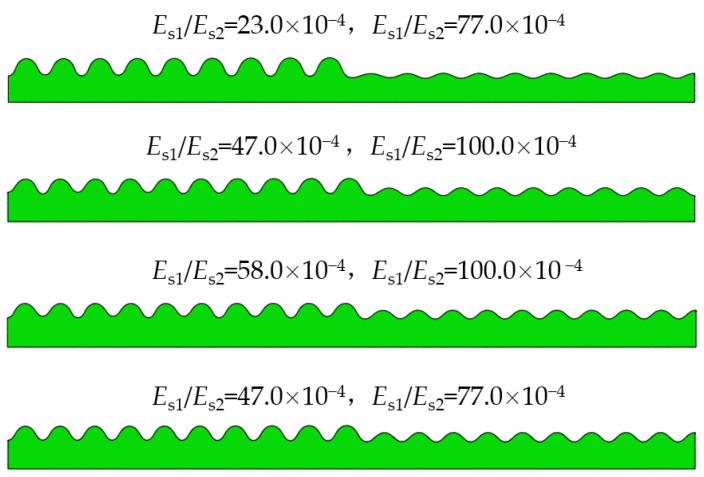
Influence of modulus ratio $E_{s1}{/E}_{s2}$ on the composite instability morphology.

The modulus ratio $E_{s1}{/E}_{s2}$ is one of the main factors that affect the wrinkling patterns of the film/substrate system. As shown in Table 2, the geometric size of the sample and the compressive strain *ε* were kept constant, and the different substrate moduli $E_{s1}$ from $6{.9\;\times \;10}^{-3}$ to $30{.0\;\times \;10}^{-3}$ were obtained by controlling the time of the first curing so that the modulus ratio $E_{s1}{/E}_{s2}$ is from $23{.0\;\times \;10}^{-4}$ to $100{.0\;\times \;10}^{-4}$. The composite bilayer system with surface wrinkling patterns obtained by varying the modulus ratio $E_{s1}{/E}_{s2}$ of the two periodic unit cells is depicted in Figure 4. When the modulus ratios are equal to $77{.0\;\times \;10}^{-4}\;$ and $23{.0\;\times \;10}^{-4}$, the composite system surface produces two sinusoidal patterns with greatly different amplitudes, with amplitudes of 6.5 mm and 1.9 mm, respectively. When the unit cell with modulus ratio $E_{s1}{/E}_{s2}$ of $47{.0\;\times \;10}^{-4}$ is combined with $77{.0\;\times \;10}^{-4}$, the amplitude of the sinusoidal wrinkle pattern increases significantly, reaching 3.2 mm. With the modulus ratio continuously changing, the amplitude variation of the surface pattern in the composite system was consistent with the above-mentioned law. The reason for the above phenomenon is that the elastic modulus of the two substrates is different during the destabilization process, which results in two kinds of mismatch stresses in the composite system and leads to the difference in the amplitude of the surface pattern. If the elastic modulus of the two substrates approaches gradually, the two surface patterns in the composite system will tend to be coincident.

**Figure 5:**
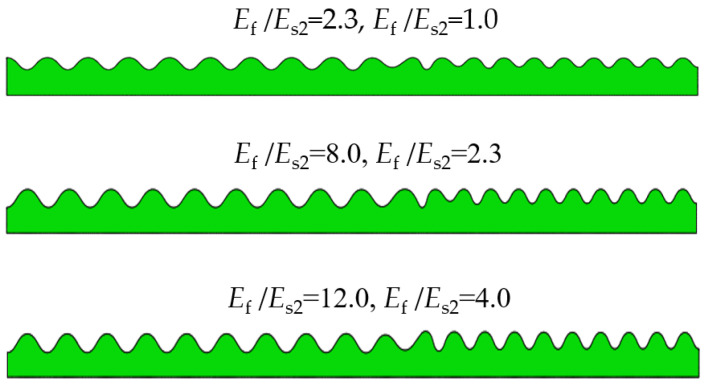
Influence of modulus ratio $E_{f}{/E}_{s2}$ on the composite instability morphology.

Similarly, the other parameters in Table 3 are kept constant except that the film modulus is taken from 3.0 to 35.5; thus, the modulus ratio, $H_{f}{/H}_{s}\;$ of 1.0 to 12.0, is obtained. As illustrated in Figure 5, the composite bilayer system with surface wrinkling patterns was obtained by varying the modulus ratio, $E_{f}{/E}_{s2}$, of the two periodic unit cells. It is clear that when the modulus ratio of the two units is equal to 2.3 and 1.0, 8.0 and 2.3, 12.0 and 4.0, respectively, only two sinusoidal patterns are formed on the surface of the composite system, and defects at the interface are also inevitable. The results show that the wrinkle pattern can be well controlled in the composite system by tuning the elastic moduli of the two films in one system.

In order to obtain the hybrid buckling topography composed of two different sinusoidal patterns, the value ranges of two different sets of parameters should be guaranteed. When two groups of systems with different thickness ratios, $H_{f}{/H}_{s}$, are selected for combination, it is necessary to ensure that the film thickness is greater than 0.5 mm and to try to ensure that the values of the modulus ratio $E_{s1}{/E}_{s2}$ of the two groups are close to each other so as to obtain a better sinusoidal wrinkle pattern. In addition, for the modulus ratio $E_{f}{/E}_{s2}$, the parameter can be selected in a large range.

### 2.3. Bandgap Structure of the Composite Bilayer System

**Figure 6:**
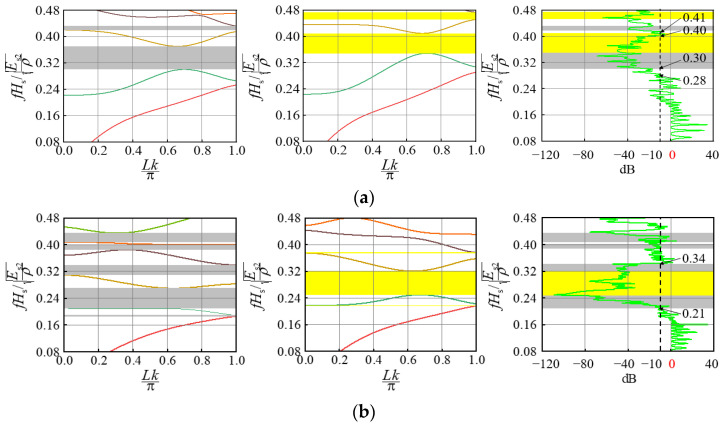
Influence of thickness ratio $H_{f}{/H}_{s}$ on the band gap: (**a**) 4.0 × 10^−2^ and 3.0 × 10^−2^; (**b**) 7.0 × 10^−2^ and 5.0 × 10^−2^.

**Figure 7:**
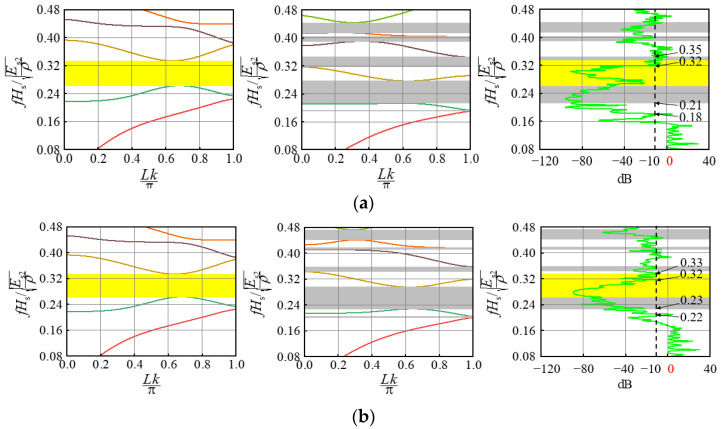
Influence of thickness ratio $E_{s1}{/E}_{s2}$ on the band gap: (**a**) 77.0 × 10^−4^ and 23.0 × 10^−4^; (**b**) 77.0 × 10^−4^ and 47.0 × 10^−4^.

**Figure 8:**
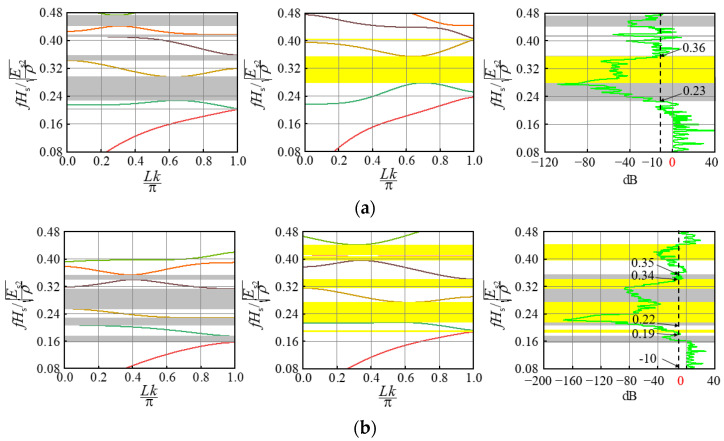
Influence of modulus ratio $E_{f}{/E}_{s2}$ on the band gap: (**a**) 2.3 and 1.0; (**b**) 12.0 and 4.0.

Depending on the geometry and material parameters of the different unit cells, surface wrinkling occurs when the composite bilayer system is compressed, resulting in composite periodic patterns. We believe that the periodic composite pattern after the substrate elastic modulus is stabilized may have a significant effect on the elastic wave propagation in the system, which is investigated in this section. It should be pointed out that Bloch wave analysis is suitable for studying the band gap of a single cell with periodic boundary conditions [20], while the transmission spectrum is suitable for studying the band gap of a composite double-layer system with limited periods. Therefore, the band gaps of two kinds of periodic cells (I and II) were studied based on Bloch wave theory, their band gap positions are shown as gray and yellow highlights in Figure 6, Figure 7 and Figure 8, respectively. And two identical periodic cell finite element mesh models were established to solve the two eigenvalue equations [24]. The frequency extraction analysis step is established in the general finite element software, ABAQUS, and then the subroutine is called on to make the wave vector, k, scan the entire irreducible Brillouin region. Finally, the energy band structure can be obtained by solving the eigenvalue with ABAQUS.

The band gaps of the composite bilayer system containing 20 periodic cells were calculated based on the transmission spectrum. In this study, Equation (2) was used to study the transport characteristics of the film-based double layer wrinkling system [24]. $u_{1\;}$ represents displacement loading excitation, and $u_{2}$ represents the dynamic response. The vibration transmission characteristic curve can be obtained through Fourier transformation and corresponding calculations. The analysis was carried out using the steady-state dynamics analysis step in ABAQUS:

(2) $$20\mathrm{lg}\;\left(\left|u_{2}/u_{1}\right|\right)$$

In the composite bilayer system shown in Figure 3, Figure 4 and Figure 5, we selected the combination with a large difference in wrinkle patterns and the combination with close wrinkle patterns for research, including a regular pattern and a defect pattern. For convenience, the wave vector was normalized as Lk/π, and the frequency was normalized as $fH_{s}/\sqrt{E_{s2}/\rho }$ [27].

For the thickness ratio, $H_{f}{/H}_{s}$, the results in Figure 6 show that when two periodic unit cells with thickness ratios of $4{.0\;\times \;10}^{-2\;}$ and $3{.0\;\times \;10}^{-2}\;$ are selected to combine, the surface of the composite system produces a sinusoidal pattern and a double period pattern. The position of the superimposed wide band gap is 0.30~0.41, while the attenuation shown by the transmission characteristic curve is 0.28~0.40. When two periodic unit cells with a thickness ratio of $7{.0\;\times \;10}^{-2}\;$ and $5{.0\;\times \;10}^{-2}\;$ are selected to combine, the period-doubling pattern disappears, and the composite instability morphology consists of two sinusoidal patterns. The position of the broadband band gap after superposition and the attenuation area shown by the vibration test is both 0.21~0.34. An important and key finding revealed is that the band gap in a composite bilayer system is largely dependent on the surface pattern in the system; when the patterns of the two groups differ greatly, the band gap width is essentially unchanged, and the overall frequency moves down. When the surface pattern difference between the two groups of the periodic unit cells in the composite system is small, the position of the band gap is basically unchanged. Therefore, adjusting the combination of the two sets of thickness ratios in the system can control the bandgap.

For the modulus ratio $E_{s1}{/E}_{s2}$, the substrate modulus dominates the entire system. When the elastic moduli of the two substrates are adjusted, the surface pattern of the composite bilayer system also changes greatly. Figure 7 reveals that when two periodic unit cell combinations with modulus ratios of $77{.0\;\times \;10}^{-4}$ and $23{.0\;\times \;10}^{-4}$ are chosen, two sinusoidal patterns with large amplitude differences are generated on the surface of the composite system. The broadband band gap position after superposition is 0.21~0.35, while the attenuation shown by the transmission characteristic curve is 0.18~0.32. When the combination of two periodic unit cells with a modulus ratio of $77{.0\;\times \;10}^{-4}$ and $47{.0\;\times \;10}^{-4}$ is selected, the position of the superimposed wide band gap is 0.23~0.33, and the attenuation shown by the transmission characteristic curve is 0.22~0.32. The results show that the width of the band gap remains unchanged after changing the elastic moduli of the two groups of substrates in the composite system. When the elastic moduli of the two groups of substrates differ greatly, the bandgap frequency of the system is shifted downward. However, when the elastic moduli of the two groups are close, the band gap frequency of the system gradually approaches the region after the superposition of the band gap frequencies of the two groups of periodic cells.

As for modulus ratio $E_{f}{/E}_{s2}$, as shown in Figure 8, when two kinds of periodic cell combinations with modulus ratios of 2.3 and 1.0 are selected, the broadband band gap after superposition is in good agreement with the attenuation area displayed by the transmission characteristic curve, both of which are 0.23~0.36. When two kinds of periodic cell combinations with modulus ratio $E_{f}{/E}_{s2}$ of 12.0 and 4.0 are selected, the broadband band gap position after superposition is 0.22~0.35, while the attenuation shown by the transmission characteristic curve is 0.19~0.34, with a certain downward shift. When the material of the film is changed, the influence on the surface pattern of the composite bilayer system is weak. The film thickness can be selected in a large range. Compared with the frequency after the superposition of the band gaps of the periodic cells, the band gap frequency and width of the composite system are basically the same. The overlap of the band gaps may cause a sudden change in the band gap attenuation so that the band gap attenuation in the overlapping region is weakened.

Compared with the band gaps of the two single-instability morphology film/substrate systems after simple superposition, when the amplitude of the pattern in the hybrid buckling morphology changes, the width of the band gap remains unchanged, but the frequency shifts down as a whole, so it is beneficial to the adjustment of the band gap. In order to obtain a wide band gap with a lower frequency, the parameters $H_{f}{/H}_{s}$ and $E_{s1}{/E}_{s2}$ can be changed, and a wide band gap with a constant width and frequency can be obtained by changing the parameter of $E_{f}{/E}_{s2}$. Additionally, the wrinkled structure was successfully prepared by a moisture-curing process, and it was confirmed by simulation and experiment that the structure has good band gap properties.

## 3. The Experiment

### 3.1. Wrinkled Sample 

**Figure 9:**
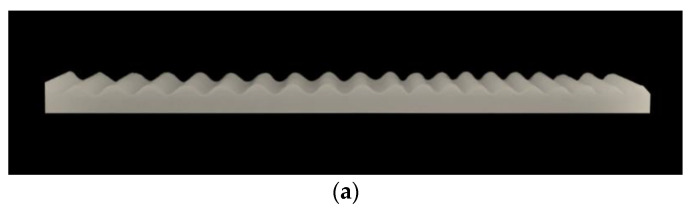

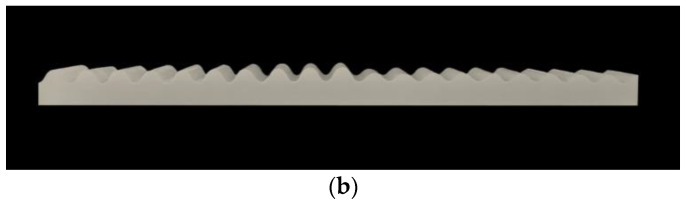
Wrinkled sample with composite patterns: (**a**) the thickness ratio $H_{f}{/H}_{s}$ of 7.0 × 10^−2^ and 5.0 × 10^−2^; (**b**) the modulus ratio $E_{s1}{/E}_{s2}$ of 77.0 × 10^−4^ and 47.0 × 10^−4^.

Previous studies have shown that it is difficult to overcome the influence of the accumulated stress in the process of instability by selecting the general elastic instability mode, and the sample may produce large deformation and lead to overall warpage. When the materials of the film and the substrate are different, it is difficult to build a high interface strength to deal with the problem of debonding. Through the optimization of the formula and the design of the moisture-curing process, as shown in Figure 9, the thickness ratio $H_{f}{/H}_{s}$ is selected to be $7{.0\;\times \;10}^{-2}$ and $5{.0\;\times \;10}^{-2}$ and periodic cells with the modulus ratio $E_{s1}{/E}_{s2}$ of $77{.0\;\times \;10}^{-4}$ and $47{.0\;\times \;10}^{-4}$, respectively. The composite bilayer system with regular surface wrinkling patterns was obtained. The substrate experimental material used for this paper was GMX-H40 silicone rubber, which was purchased from the China Bluestar Chengrand Research Institute of Chemical Industry (Chengdu, China). The silicone rubber has a white paste appearance and has the advantages of good fluidity, low shrinkage, good dimensional stability, and aging resistance, and it is widely used in mold forming and manufacturing, sealing and filling, and other fields.

### 3.2. Vibration Test

#### 3.2.1. Device

**Figure 10:**
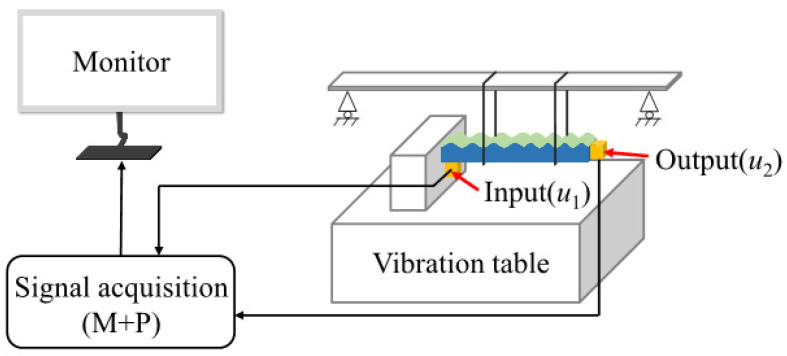
Schematic diagram of vibration test system.

**Figure 11:**
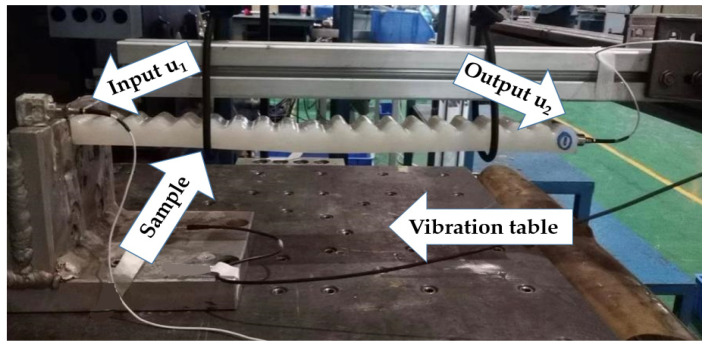
Vibration test setup.

In order to evaluate the band gap performance of the composite double-layer sample, a vibration test was carried out on the vibration test system. Figure 10 is a schematic diagram of the vibration test system. The system is mainly composed of an excitation system and a data acquisition system. The purpose of hanging the sample on the shaker was to eliminate the friction between the bottom of the sample and the vibration table. The left end face of the sample was fixed with the right end face of the iron block, an acceleration sensor for receiving the input ($u_{1}$). The vibration signal was installed on the left end face of the sample, and then an acceleration sensor was installed on the right end face of the sample for receiving the output ($u_{2}$) vibration signal. The picture of the real experimental setup is shown in Figure 11. The sample was excited by a sinusoidal frequency sweep with a shaking table, and the acceleration of the excitation point and response point was measured. The sample was excited by a sine frequency sweep using a shaking table, and the acceleration of the excitation point and the response point was measured, and then the vibration transmission characteristic curve was obtained through Fourier transform (FRF) and corresponding operations.

#### 3.2.2. Test Results

**Figure 12:**
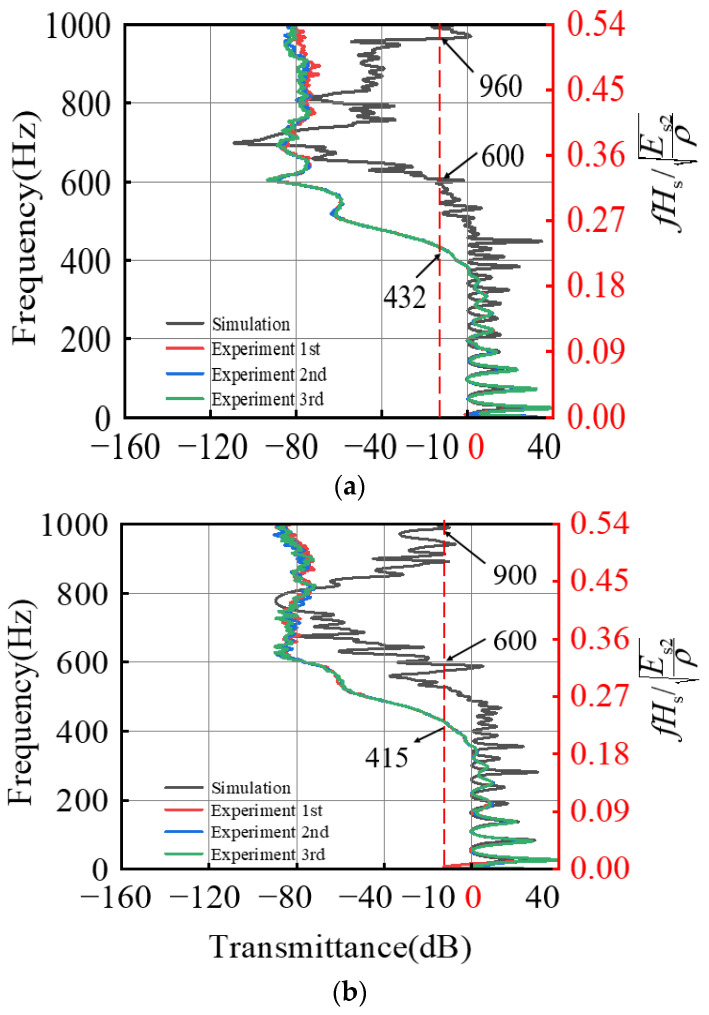
Vibration transmission characteristic curve: (**a**) the thickness ratio $H_{f}{/H}_{s}$ of 7.0 × 10^−2^ and 5.0 × 10^−2^; (**b**) the modulus ratio $E_{s1}{/E}_{s2}$ of 77.0 × 10^−4^ and 47.0 × 10^−4^.

Transmission spectra were defined as $20\mathrm{lg}\;\left(\left|u_{2}/u_{1}\right|\right)$, which were used to evaluate the band gap properties of the samples being tested. This work focuses on band gap characteristics below 1000 Hz, which are often used to control the propagation of elastic waves in the low-frequency range. Figure 12 shows the test results in the frequency range of 0 to 1000 Hz. The experimental results show that the transmission characteristic curves obtained by the vibration test are basically consistent at the initial frequency, indicating that the composite wrinkling bilayer system can effectively superimpose the band gap structures corresponding to two different periodic unit cells. The composite wrinkling system we constructed has wide band gap characteristics. However, the attenuation range is significantly larger than that obtained by numerical simulation. This is because the base modulus is too small, and the whole sample acts as a damping material so that the attenuation strength of the vibration signal increases with the increase of frequency.

## 4. Discussion

When designing composite bilayer systems with wide band gaps, different parameters can be selected, including thickness ratio $H_{f}{/H}_{s}$ and modulus ratio $E_{f}{/E}_{s2}$. In order to obtain a band gap that is completely consistent with the superposition of periodic cells, select a parameter that has little influence on the wrinkle pattern, such as $E_{s1}{/E}_{s2}\;$. If you want to design a wide band gap that is lower than the frequency after the superposition of the band gaps of the periodic cells, you can design defects; that is, select $H_{f}{/H}_{s}$ and $E_{s1}{/E}_{s2}$ for the design.

**Figure 13:**
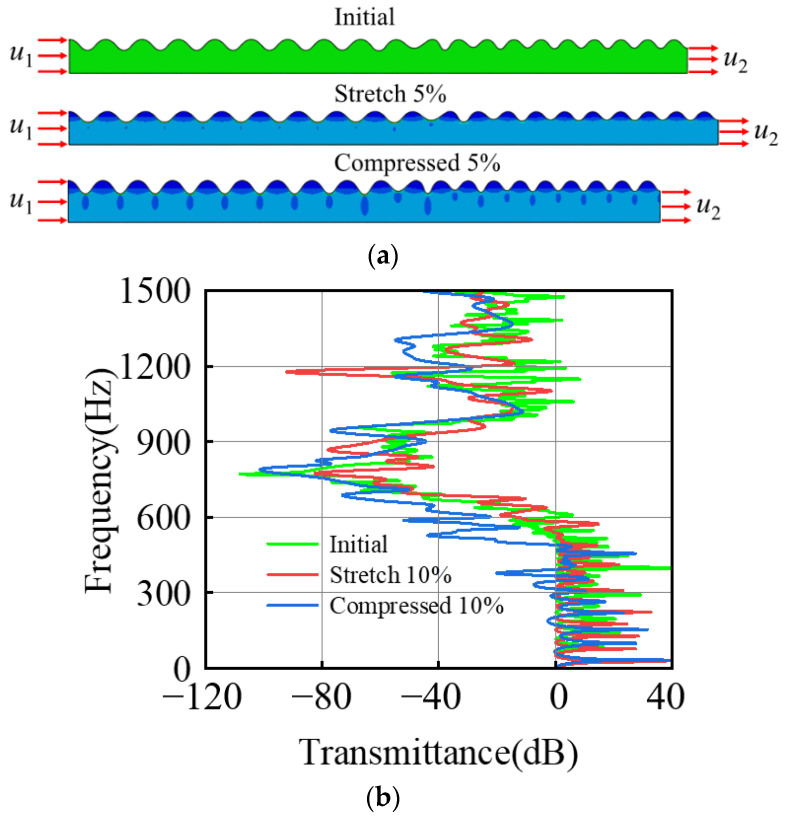
Influence of a 5% external load on the system: (**a**) deformation under a 5% tensile and compressive load; (**b**) transmission characteristic curve.

In order to explore the effect of external load on the band gap of a composite bilayer system, a composite system composed of two groups of periodic cells with a thickness ratio $H_{f}{/H}_{s}$ of $4{.0\;\times \;10}^{-2}$ and $3{.0\;\times \;10}^{-2}$ should be selected for research. When a 5% tensile load and a 5% compressive load are applied, respectively, it can be seen that there is no obvious difference in the attenuation region, indicating that a small range of strain has little effect on the band gap frequency, as shown in Figure 13.

**Figure 14:**
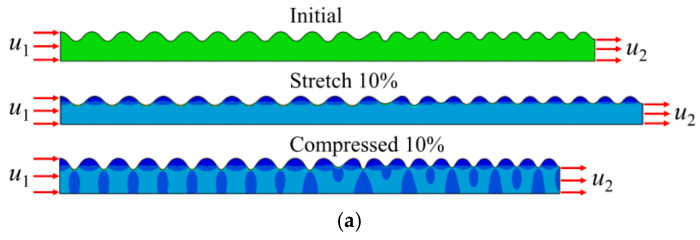

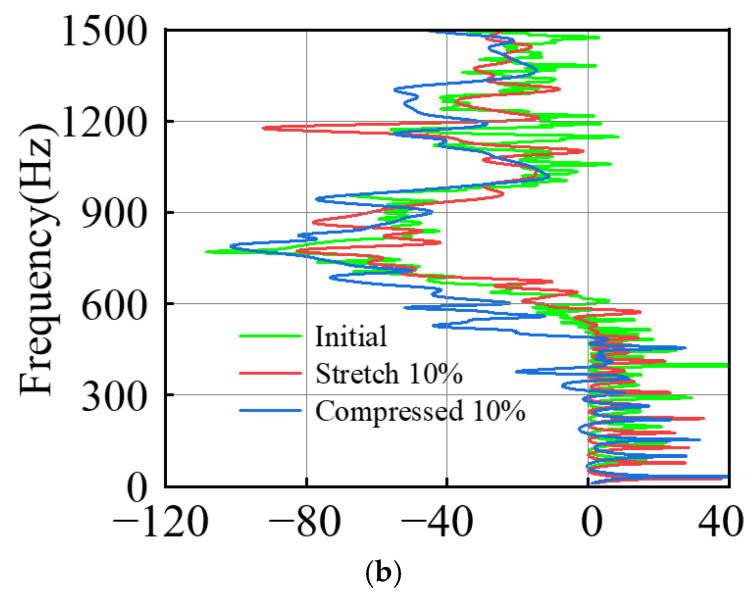
Influence of a 10% external load on the system: (**a**) deformation under a 10% tensile and compressive load; (**b**) transmission characteristic curve.

As shown in Figure 14, the attenuation frequency range decreases when tensile loads of 10% are applied. When the system is compressed by 10%, the attenuation range increases; that is, the band gap width increases. Therefore, an external strain can regulate the band gap width of the wrinkling system, and the magnitude of the tension or compression will be determined according to the actual demand.

## 5. Conclusions

A wrinkling bilayer system with two wrinkle patterns and a wide band gap has been reported. The system can control the width and frequency of the band gap structure by changing the pattern wavelength and shape. When the thickness ratio $H_{f}{/H}_{s}$ is less than 0.03, the period doubling patterns appear in the hybrid instability system, while the band gap width is essentially unchanged, and the overall frequency moves down. When the elastic modulus ratio $E_{s1}{/E}_{s2}$ of the two groups of substrates differ greatly, the bandgap frequency of the system is shifted downward. When the modulus ratio $E_{f}{/E}_{s2}\;$ of the two selected films is quite different, the band gap of the hybrid system moves down. In general, hybrid systems can effectively stack two bandgaps and therefore expand the band gap structure.

For different engineering application scenarios, the band gap characteristics of the wrinkling system under different loading conditions were considered. The results show that when the load is 5%, the small deformation of the system pattern makes the band gap characteristics have good stability. When the load is 10%, the large deformation of the system pattern makes the band gap range shift. Considering the variation of the frequency attenuation region of the hybrid system under different external loads may provide a design idea for vibration control in practical application scenarios. The hybrid system created by combining two groups of film/substrate wrinkling patterns can be used to gain wide attenuation frequency ranges to control the elastic wave propagation.

## Figures and Tables

**Table 1:** Different thickness ratios, $H_{f}{/H}_{s}$.

Thickness Ratio $H_{f}{/H}_{s}$	Compressive Strain ε	Film Thickness $H_{f}$ (mm)	Substrate Thickness $H_{s}$ (mm)	Film Modulus $E_{f}$ (MPa)	Substrate Modulus $E_{s1}$ (MPa)	Substrate Modulus $E_{s2}$ (MPa)
3.0 × 10^−2^	20%	0.6	18.0	6.9	13.8 × 10^−3^	3.0
4.0 × 10^−2^	20%	0.7	18.0	6.9	13.8 × 10^−3^	3.0
5.0 × 10^−2^	20%	0.9	18.0	6.9	13.8 × 10^−3^	3.0
6.0 × 10^−2^	20%	1.0	18.0	6.9	13.8 × 10^−3^	3.0
7.0 × 10^−2^	20%	1.2	18.0	6.9	13.8 × 10^−3^	3.0

**Table 2:** Different elastic modulus ratios, $E_{s1}{/E}_{s2}$.

Modulus Ratio $E_{s1}{/E}_{s2}$	Compressive Strain ε	Film Thickness $H_{f}$ (mm)	Substrate Thickness $H_{s}$ (mm)	Film Modulus $E_{f}$ (MPa)	Substrate Modulus $E_{s1}$ (MPa)	Substrate Modulus $E_{s2}$ (MPa)
23.0 × 10^−4^	20%	1.0	18.0	6.9	6.9 × 10^−3^	3.0
47.0 × 10^−4^	20%	1.0	18.0	6.9	13.8 × 10^−3^	3.0
58.0 × 10^−4^	20%	1.0	18.0	6.9	17.2 × 10^−3^	3.0
77.0 × 10^−4^	20%	1.0	18.0	6.9	23.0 × 10^−3^	3.0
100.0 × 10^−4^	20%	1.0	18.0	6.9	30.0 × 10^−3^	3.0

**Table 3:** Different elastic modulus ratios, $E_{f}{/E}_{s2}$.

Modulus Ratio $E_{f}{/E}_{s2}$	Compressive Strain ε	Film Thickness $H_{f}$ (mm)	Substrate Thickness $H_{s}$ (mm)	Film Modulus $E_{f}$ (MPa)	Basement Modulus $E_{s1}$ (MPa)	Basement Modulus $E_{s2}$ (MPa)
1.0	20%	1.0	18.0	3.0	13.8 × 10^−3^	3.0
2.3	20%	1.0	18.0	6.9	13.8 × 10^−3^	3.0
4.0	20%	1.0	18.0	11.8	13.8 × 10^−3^	3.0
8.0	20%	1.0	18.0	23.7	13.8 × 10^−3^	3.0
12.0	20%	1.0	18.0	35.5	13.8 × 10^−3^	3.0

## Data Availability

Some or all of the data, models, or code generated or used during the study are available from the corresponding authors by request.
